# iTRAQ-based quantitative proteomic analysis of Yamanaka factors reprogrammed breast cancer cells

**DOI:** 10.18632/oncotarget.16125

**Published:** 2017-03-11

**Authors:** Kun Wang, Zhiyan Shan, Lian Duan, Tiantian Gong, Feng Liu, Yue Zhang, Zhendong Wang, Jingling Shen, Lei Lei

**Affiliations:** ^1^ Department of Histology and Embryology, Harbin Medical University, Harbin, China; ^2^ Embryo and Stem Cell Engineering Laboratory, Harbin Medical University, Harbin, China; ^3^ College of Bioinformatics Science and Technology, Harbin Medical University, Harbin, China; ^4^ Department of Breast Surgery, Cancer Hospital of Harbin Medical University, Harbin, China

**Keywords:** breast cancer, reprogramming, iTRAQ, proteome, mitochondria

## Abstract

Cancer cells had been developed to be reprogrammed into embryonic stem like cells by induced pluripotent stem cells (iPSCs) technology, however, the tumor differentiation/dedifferentiation mechanisms had not yet been analyzed on a genome-wide scale. Here, we inserted the four stem cell transcription factor genes OCT4, SOX2, C-MYC and KLF4 into MCF cells (MCFs), represented a female breast cancer cell type, and obtained iPSCs (Mcfips) in about 3 weeks. By using the LC MS/MS iTRAQ technology, we analyzed the proteomic changes between MCFs and Mcfips. Of identified 4,616 proteins totally, 247 and 142 differentially expressed (DE) proteins were found in Mcfips compared with human induce pluripotent stem cells (Hips) and MCFs, respectively. 35 co-up and 10 co-down regulated proteins were recognized in DE proteins. Above DE proteins were categorized with GO functional classification annotation and KEGG metabolic pathway analysis into biological processes. In the protein interaction network, we found 37 and 39 hubs interacted with more than one protein in Mcfips comparing to Hips, in addition, 25 and 9 hubs were identified in Mcfips comparing to MCFs. Importantly, the mitochondria, ribosome and tumor suppressor proteins were found to be core regulators of tumor reprogramming, which might contribute to understand the mechanisms in relation to the occurrences and progression of a tumor. Thus, our study provided a valuable data for exploring the possibility to normalize the malignant phenotype.

## INTRODUCTION

Breast cancer (BC) is the most common cancer and the second leading cause of cancer death in women [1, 2]. Along with the proceeding of age, the incidence rate of BC showed an upward trend. Since limited understanding of occurrence and development, there was no effective treatment for breast cancer besides operation. New concepts and methods must be introduced and produced new breakthroughs.

In 2006, Takahashi and Yamanaka successfully reprogrammed somatic cells to induced pluripotent stem cells (iPSCs), which brought new opportunities for pathogenetic mechanism studies and specific therapy for tumor [3]. Since somatic cells could be completely reversed into iPSCs, in theory, the tumor cells could also be reprogrammed. Current studies have shown that tumor cells could be induced by iPS technology, and tumor cells, despite the presence of oncogenic mutations, acquired pluripotency and underwent differentiation into cell types derived of all 3 germ layers during teratoma formation [4, 5]. Some results also demonstrated the induced cells were iPSCs, which were distinct from natural cancer cells with regard to their sensitivity to differentiation-inducing treatment [6–8]. However, whether the tumor induced cells had the characteristics of stem cell or tumor, or both, was still not determined. Moreover, the transformation mechanism between stem cells and tumor cells was not clear. Recent research showed that the procession of tumor was related to the degree of tumor differentiation, and the lower degree of differentiation of the tumor was more likely to express stem cell-related genes, such as Oct4, Sox2, Nanog, and so on, but these genes were not necessarily expressed at the same time [6]. Finding the expression patterns of genes could be beneficial for study of the transformation between cancer and stem cell.

It could be speculated that iPS technology would play an important role and have a profound impact on the prevention and treatment of BC. However, there were few researches that used iPSCs to investigate the mechanism of occurrence, invasion and metastasis of BC. In this study, by using the LC MS/MS iTRAQ technology, we determined the DE proteins between BC-iPSCs (Mcfips) and human iPSCs (Hips) to study the regulation mechanism of tumor cell reprogramming. Then we compared the Mcfips with BC cells (MCFs) to obtain the changes of tumor related genes during reprogramming. Therefore, combining iPS technology and bioinformatics analysis, we could capture and select the important signaling molecules during tumor cell reprogramming. Further analysis of signaling networks could be helpful to understand the transform mechanism between tumor and stemness, which provided a basis for searching new targets for tumor therapy.

## RESULTS

### Introducing the reprogramming factors into a breast cancer cell line

To address whether human BC cells could be reprogrammed into iPSCs, MCFs were transfected by ecotropic retrovirus with four reprogramming factors, OCT3/4, SOX2, KLF4, and c-MYC at day 0. We added the HDAC inhibitor, VPA, to improve reprogramming efficiency, which had been showed much stronger than other HDAC inhibitors during reprogramming. Six days after transduction, the cells were harvested by trypsinization and plated onto feeder cells. Ten days later, some colonies appeared that were morphologically different from the parental cancer cells (Supplementary Figure 1). More embryonic stem (ES)-like colonies emerged 20 days after the virus infection and were picked for expansion (Supplementary Figure 1A). The isolated iPSCs were morphologically very similar to hES cells, which exhibiting tightly packed colonies with a high nucleocytoplasmic ratio and refractive edges. Immunofluorescence analysis showed that these Mcfips expressed TRA-1-81 and NANOG, suggesting that Mcfips were indistinguishable from the ES-like cells (Supplementary Figure 1B).

### DE Proteins in Mcfips comparing to Hips and MCFs

**Figure 1:**
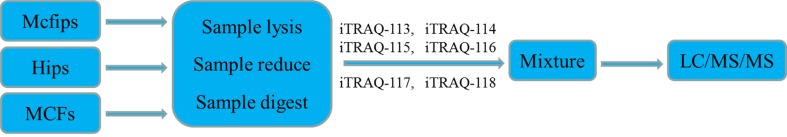
Schematic flowchart of the iTRAQ method

**Figure 2:**
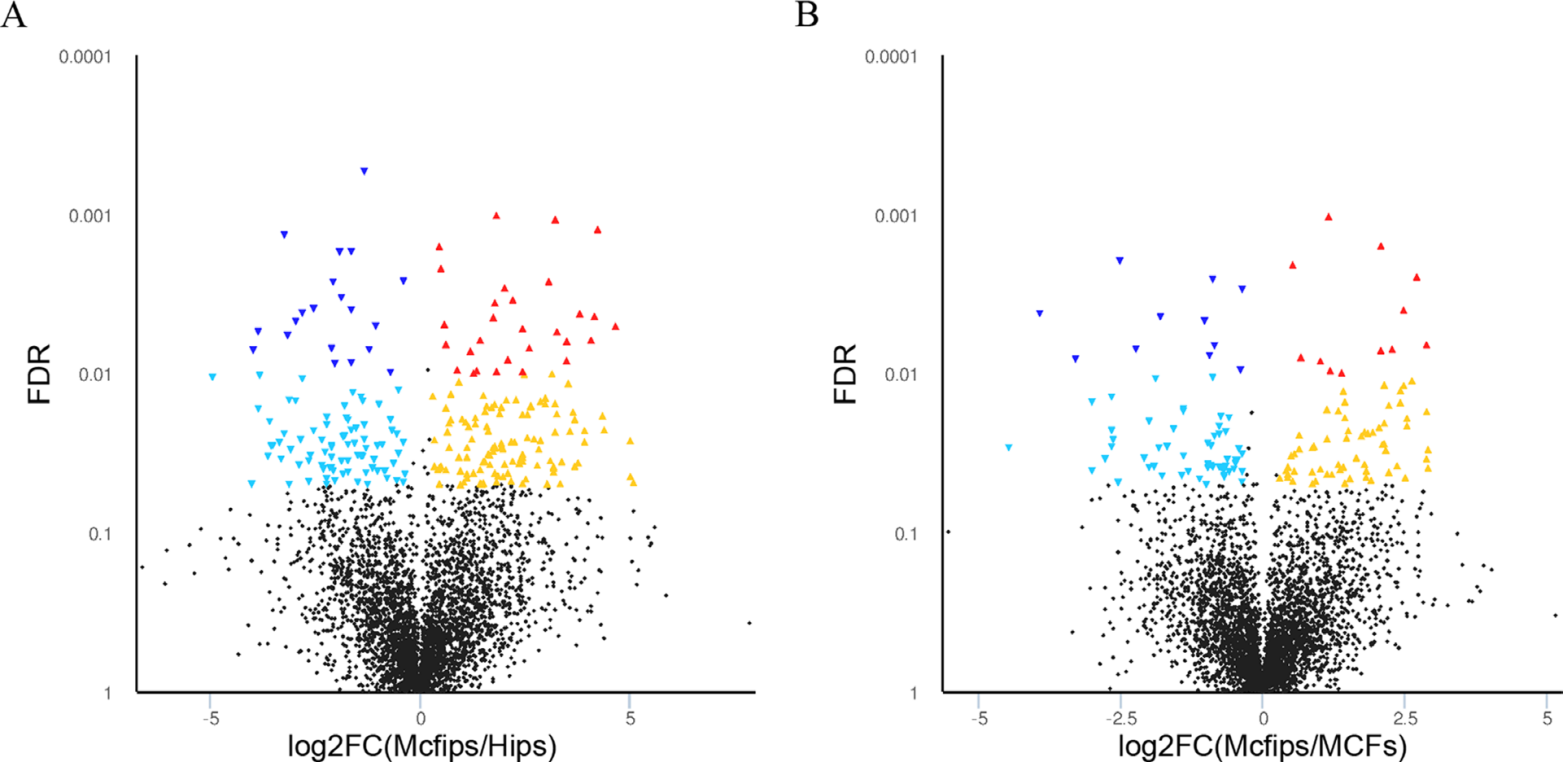
Volcano plot of proteins expressed in the Mcfips comparing to MCFs and Hips, respectively The X-axis represents log2-fold change of high- (positive values) and low-expressed (negative values) proteins in (**A**) Mcfips comparing in comparison to Hips, (**B**) Mcfips comparing in comparison to MCFs. The Y-axis corresponds to the false discovery rate (FDR) of this fold change. Blue inverted triangle: low-expressed protein with FDR < 0.01; green inverted triangle: low-expressed protein with FDR < 0.05; black spot: no significant differential protein; red triangle: high-expressed protein with FDR < 0.01; yellow triangle: high-expressed protein with FDR < 0.05.

To determine the protein expression profiles of Mcfips in comparison to Hips and MCFs, respectively, we used the approach of iTRAQ labelling and tandem mass spectrometry. The schematic flowchart of iTRAQ method was shown in Figure 1. Over 4,616 proteins were quantified with two or more peptides from six samples (two MCFs, two Mcfips, and two Hips samples). Protein lists were exported to PDST (ProteinPilot Descriptive Statistics Template) template to analyze the false discovery rate (FDR) and proteins plotted according to their log fold change (FC) and FRD-values as a volcano plot (Figure 2).

**Figure 3:**
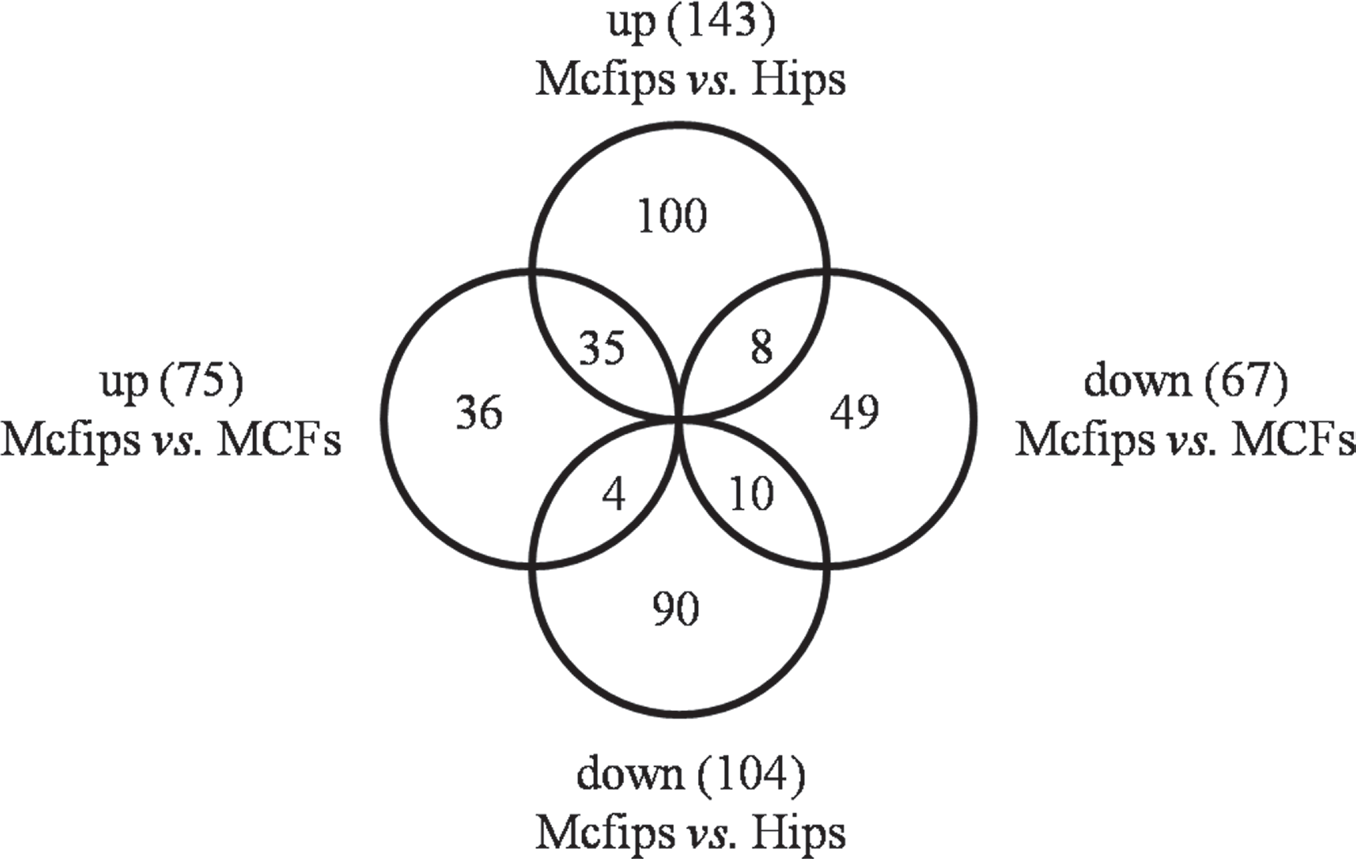
Venn-Euler diagrams of differentially expressed proteins in Mcfips comparing to Hips and MCFs

Employing this strategy, 143 up- and 104 down-regulated proteins in Mcfips were found when compared against Hips (Supplementary Table 1). There were 67 protiens that were increased in Mcfips compared with MCFs, and 75 proteins that were decreased (Supplementary Table 2). Then we found 35 co-up and 10 co-downregulated proteins in Mcfips comparing to Hips and MCFs. The expression of eight DE proteins were higher in Mcfips than in Hips, but lower than that in MCFs. Four DE proteins whose expression values were less in Mcfips than in Hips, and also were more in Mcfips than in MCFs (Figure 3, Supplementary Table 3).

### Functional annotation of the DE proteins in Mcfips comparing to Hips and MCFs

In order to obtain a global functional view of the DE proteins, Gene Ontology (GO) functional classification annotation and Kyoto Encyclopedia of Genes and Genomes (KEGG) metabolic pathway analysis were employed. The GO functional annotation analysis in Mcfips comparing to Hips and MCFs was performed including biological process (BP), molecular function (MF), and cellular component (CC).

**Figure 4:**
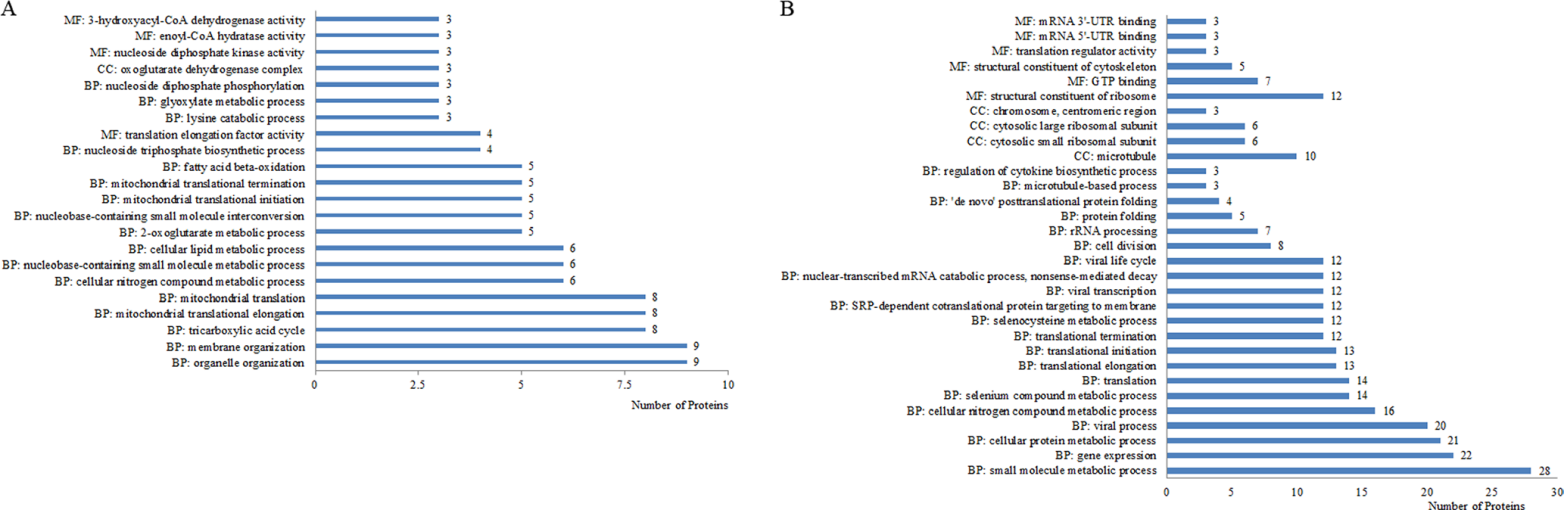
Up- (**A**) and down-regulated proteins (**B**) categorized by biological process (BP), molecular function (MF), and cellular component (CC) in Mcfips comparing to Hips.

**Figure 5:**
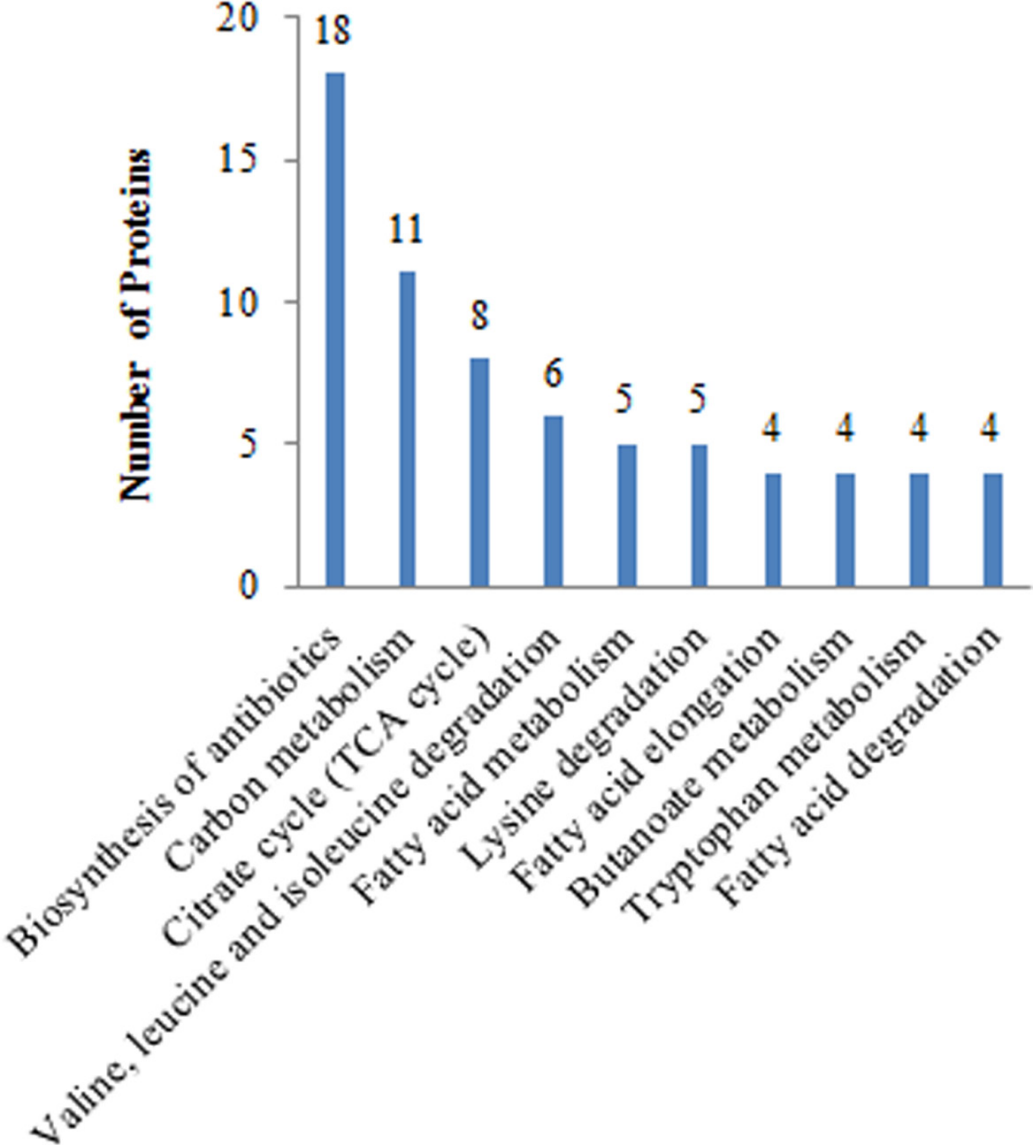
Global view of the KEGG pathways affected in Mcfips comparing to Hips

In the comparison group of Mcfips and Hips, the proteins with increased expression representing BPs included organelle (6%), membrane organization (6%), tricarboxylic acid cycle (5%), mitochondrial translational elongation (5%), mitochondrial translation (5%), and etc.. The MFs categories were translation elongation factor activity (3%), nucleoside diphosphate kinase activity (2%), enoyl-CoA hydratase activity (2%), and 3-hydroxyacyl-CoA dehydrogenase activity (2%). The proteins representing CCs were classified as oxoglutarate dehydrogenase complex (2%) (Figure 4A). The proteins with decreased expression were categorized as BPs, MFs, and CCs according to the GO database. Top five BP proteins represented small molecule metabolic process (25%), gene expression (20%), cellular protein metabolic process (19%), viral process (18%), and cellular nitrogen compound metabolic process (14%). Top three MFs proteins were also classified into the categories included structural constituent of ribosome (11%), GTP binding (6%), and structural constituent of cytoskeleton (4%). Identified CC proteins were classified as belonging to the microtubule (9%), cytosolic small ribosomal subunit (5%), cytosolic large ribosomal subunit (5%) and chromosome, centromeric region (3%) (Figure 4B). The DE proteins were further defined based on KEGG. The proteins were mapped to KEGG pathways based on their KEGG gene ID. The DE proteins for up- and down-regulated were involved in ten and three KEGG pathways, respectively. For up-regulated proteins, the top two significant (*P* < 0.05) pathways were Biosynthesis of antibiotics and Carbon metabolism (Figure 5). The two significant (*P* < 0.05) pathways for down-regulated proteins were Pathogenic Escherichia coli infection and Gap junction.

In the comparison group of Mcfips and MCFs, the high-regulated proteins were categorized as BPs, MFs, and CCs according to the GO database. Top six BP proteins represented organelle organization (12%), mitochondrial translational elongation (11%), mitochondrial translation (11%), mitochondrial translational initiation (9%), mitochondrial translational termination (9%), and respiratory electron transport chain (9%). The MFs categories were structural constituent of ribosome (10%), RNA binding (9%), and NADH dehydrogenase (ubiquinone) activity (4%). The proteins representing CCs were classified as mitochondrial ribosome (3%), ribosome (3%), and mitochondrial respiratory chain complex I (3%) (Supplementary Figure 2A). The low-regulated proteins representing BPs included protein phosphorylation (8%), regulation of apoptotic process (7%), and epidermal growth factor receptor signaling pathway (7%). The proteins representing MFs were classified as ATP binding (16%), protein serine/threonine kinase activity (10%), and protein kinase activity (5%) (Supplementary Figure 2B). The DE proteins for up-regulated were 12 KEGG pathways, and the significant (*P* < 0.05) pathways were Biosynthesis of antibiotics, Carbon metabolism, Oxidative phosphorylation, Parkinson's disease, Huntington's disease, Alzheimer's disease, Non-alcoholic fatty liver disease (NAFLD), Valine, leucine and isoleucine degradation, Citrate cycle (TCA cycle), Pyruvate metabolism, Fatty acid degradation, and Fatty acid metabolism (Supplementary Figure 3). There were two significant pathways (*P* < 0.05) for down-regulated proteins that were Regulation of Regulation of actin cytoskeleton and Focal adhesion.

We also identified 35 co-up and 10 co-down regulated proteins in Mcfips comparing to Hips and MCFs. The GO functional annotation analysis of 35 co-up regulated proteins found that they represented BPs included generation of precursor metabolites and energy (23%), generation of precursor metabolites and energy (23%), oxidation reduction (19%), oxidation reduction (19%), and translation (16%), represented MFs were classified as cofactor binding (13%), coenzyme binding (10%), and structural constituent of ribosome (10%). Top two CCs proteins represented mitochondrial part (48%), and mitochondrial part (48%) (Supplementary Figure 4). There were three significant pathways (*P* < 0.05) of 35 co-up regulated proteins including Parkinson's disease, Oxidative phosphorylation, and Huntington's disease. The GO functional annotation analysis of 10 co-down regulated proteins found that they represented MFs included metal ion (27%), cation (27%), and ion binding (27%).

### Protein-protein interaction networks of the DE proteins in Mcfips comparing to Hips and MCFs

**Figure 6:**
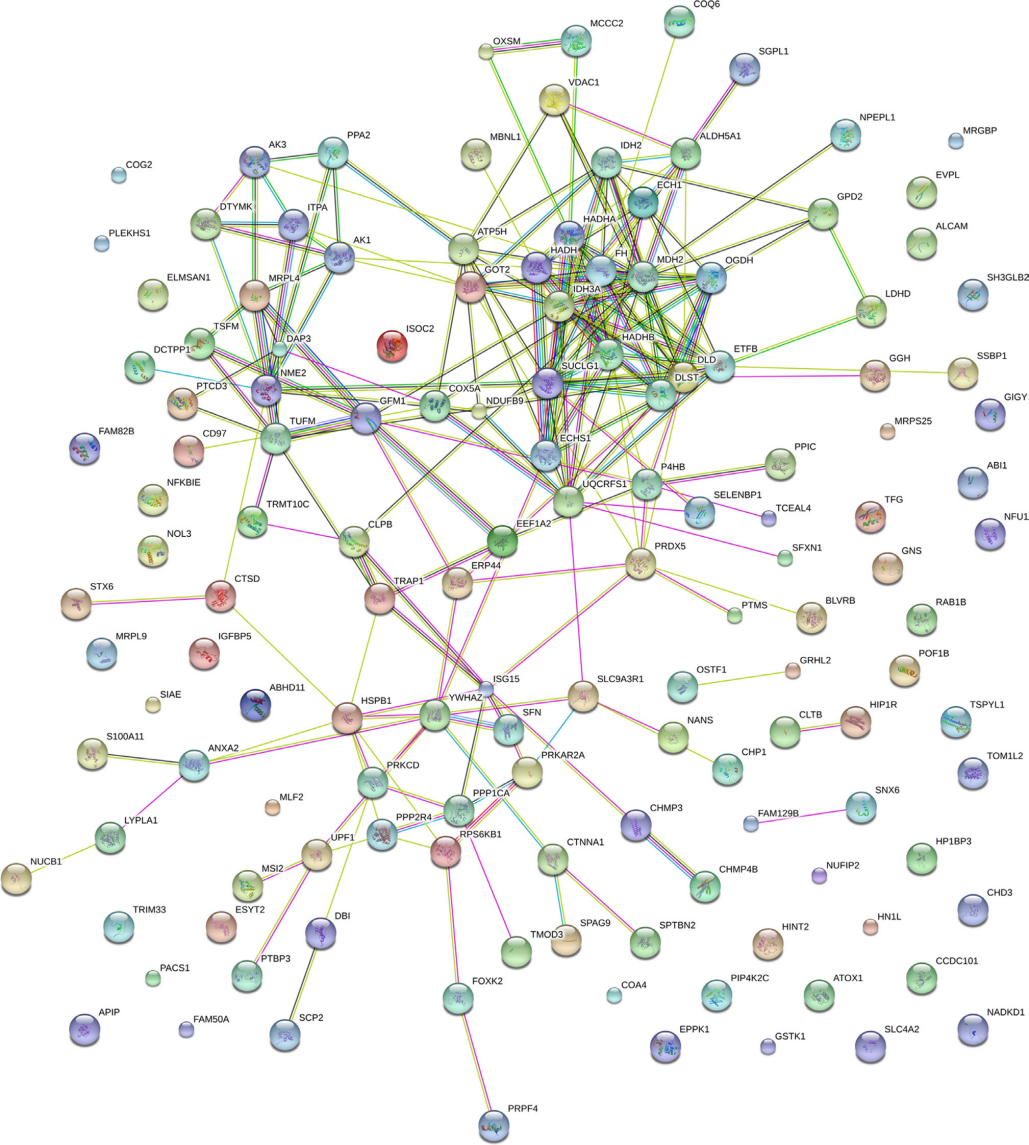
Interactome network of up-expressed proteins in Mcfips comparing to Hips Coloured lines represent different evidences for each interaction: red line, fusion; green line, neighbourhood; blue line, cooccurrence; purple line, experimental; yellow line, text mining; light blue line, database; black line, coexpression.

**Figure 7:**
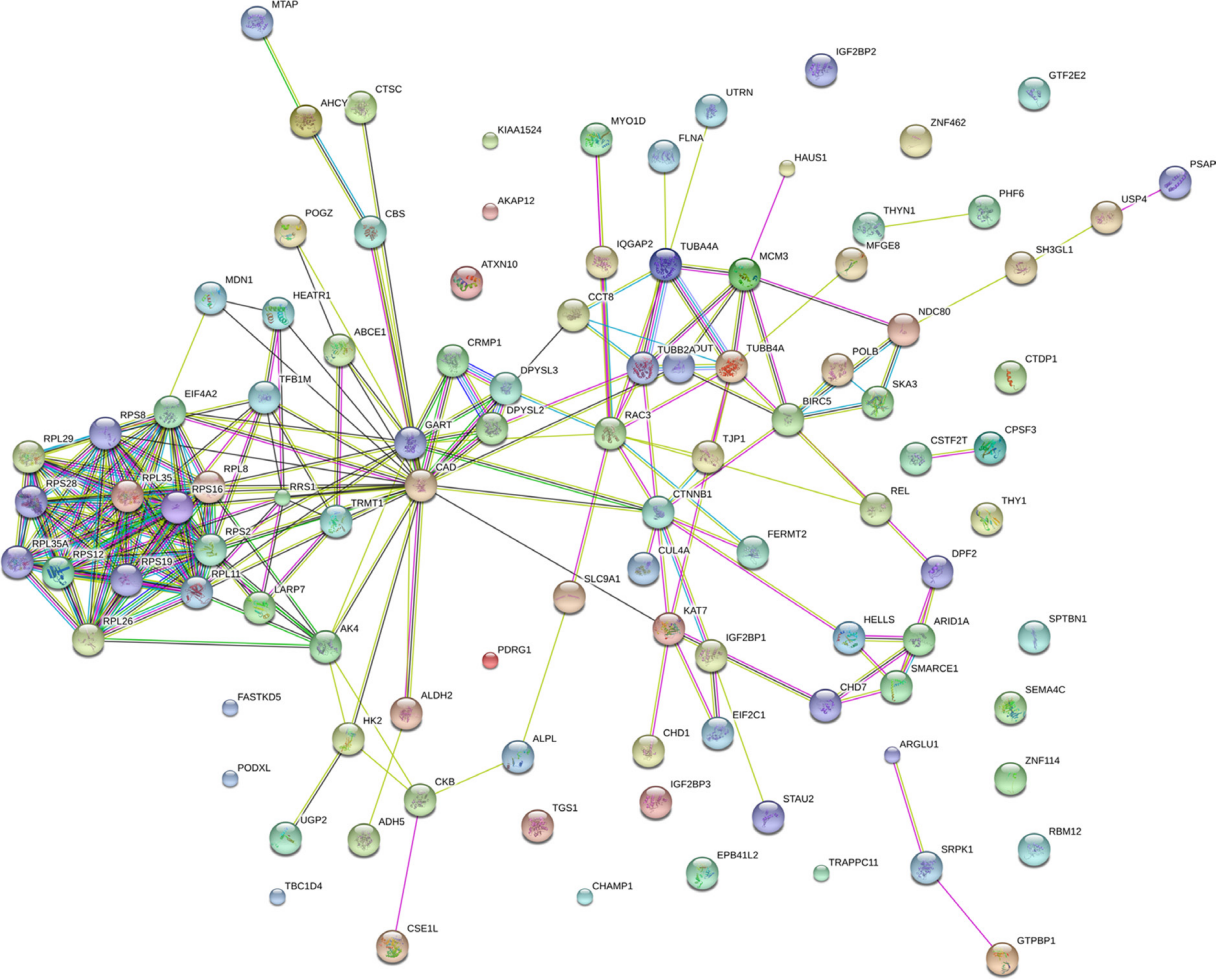
Interactome network of down-expressed proteins in Mcfips comparing to Hips Coloured lines represent different evidences for each interaction: red line, fusion; green line, neighbourhood; blue line, cooccurrence; purple line, experimental; yellow line, text mining; light blue line, database; black line, coexpression.

To observe the network of protein-protein interactions between the DE proteins in Mcfips comparing to Hips and MCFs, a network was performed with the Cytoscape software. We obtained the key nodes by calculating the statistical network measures that included Degree Centrality, Betweenness, Closeness, and Cluster Coefficient. The interaction network took proteins as its nodes, and assigned an edge between two proteins if they interacted with one another. These interactions contained direct (physical) and indirect (functional) interactions, derived from numerous sources such as experimental repositories or computational prediction methods. In the protein interaction network of up-regulated protein of Mcfips comparing to Hips, there were 37 hubs that were discovered interacted with more than one protein (Figure 6). Among those, SUCLG1, FH and MDH2 were interacted with fourteen, twelve and eleven proteins, respectively. In the protein interaction network of down-regulated protein between Mcfips and Hips, 39 hubs were recognized that were interacted with more than one protein (Figure 7). Among those, GART were interacted with fifteen proteins, RPS19 and RPS28 were interacted with twelve proteins together. In Mcfips comparing to MCFs, 25 hubs and 9 hubs were interacted with more than one protein in the up- (Supplementary Figure 5) and down-regulated protein network (Supplementary Figure 6), respectively. MRPS14 and TSFM were interacted with twelve proteins in the up-regulated protein network. And in the down-regulated protein network, ACTN1 and SPTAN1 were interacted with seven and six proteins, respectively.

And in the protein interaction network of 35 co-up regulated proteins in Mcfips comparing to Hips and MCFs, there were 16 hubs that were identified interacted with more than one protein (TUFM, TSFM, MRPL4, DLD, OGDH, SUCLG1, NDUFB9, UQCRFS1, MDH2, ATP5H, GOT2, VDAC1, COX5A, AK1, PPA2, and HADH, Supplementary Figure 7). Among those, MDH2 and ATP5H were identified interacted with eight and seven proteins, respectively. In the protein interaction network of 10 co-down regulated proteins, SH3GL1 and USP4 were identified interacted with each other (Supplementary Figure 8).

## DISCUSSION

Previous reported showed that both normal and tumor tissues could be reprogrammed by iPSCs technology, which contributed to better understand development and progression. However, the derived iPSCs in different group displayed critical differences in biological behaviors, such as pluriotency and malignancy. Some tumors could be reprogrammed to embryonic stem like cells with typical stem cell markers, and others were changed to be caner stem cells with enhancing the oncogenic potential or mesenchymal stem like cells that lost their tumorigenicity. Therefore, each tumor might be the discrepant reprogramming. Here we showed that MCFs could be reprogrammed by lentivirus infection of four Yamanaka factors. The morphology of Mcfips was similar as hiPSCs and expressed the specific marker of hiPSCs.

Taking advantage of LC MS/MS iTRAQ technology, we found 247 (5.4%) DE proteins between Mcfips and Hips, and 67 up- and 75 down-regulated proteins in Mcfips comparing with MCFs, which suggested that Mcfips might be partly reprogrammed. Furthermore, we found 11 DE proteins (FC > 8) between Mcfips and Hips, including ALCAM, ABHD11, ETFB, EPPK1, ECHS1, HINT2, HADHB, IGFBP5, IDH2, SFN, SUCLG1. Meanwhile, 11 DE proteins (FC > 5) were identified in overlap between the Mcfips and MCFs. From the two groups of date, interesting, 73% DE proteins were also related to mitochondrial function, which suggested that mitochondria might play important roles in tumor reprogramming, which was rarely reported before.

Some studies indicated that mitochondria played important roles in somatic cells reprogramming, with the decreased number of mitochondria and the increased number of immatue spherical cristae, the oxidative metabolism had been converted to glycolysis during reprogramming [9, 10]. Regulation of mitochondria biosynthesis would be benefit to the fully reprogramming of tumor. Moreover, MDH2 was identified as an interaction factor with other proteins in DE proteins. MDH2 could regulate the mitochondrial NADH/NAD+ redox state to support ATP production, the expression of MDH2 possibly reflected metabolic reprogramming of mitochondria and correlate with tumor cell proliferation. So, it might improve the tumor reprogramming process by regulating the expression of MDH2.

In addition, we also screened the DE proteins that were up regulated between Mcfips and Hips, and down regulated between Mcfips and MCFs. We found that these proteins were mainly associated with ribosome function, such as RPL11 and RPL29 [11, 12]. It indicated that nuclear proteins might play important regulatory roles in the reprogramming of tumor cells. In an attempt to get the fully reprogrammed of iPSCs, we might need to stabilize the high expression of ribosomal protein. In our study, we also discovered abnormal expression of some tumor suppressor genes, such as IGFBP5 and SFN, which high expressed in the Mcfips, but low in Hips. Previous studies demonstrated that a loss of tumor suppressor function was associated with the efficient induction of pluripotency. It was recently shown that IGFBP5 overexpression induced cell senescence in a p53-dependent manner. The p53 pathway has been identified as one primary barrier to reprogramming. Moreover, overexpression of *Igfbp5* in MEFs strongly reduced reprogramming [13] . Thus, according to previous reports and our own studies, decreased expression of IGFBP5 was likely to be beneficial to iPSC generation.

Therefore, our data mainly provided three important aspects related to tumor reprogramming, namely, mitochondria, ribosome participation and tumor suppressor genes. Armed with these dates, we might obtain the fully reprogrammed iPSCs from the tumor by regulating these genes and mechanisms, which would be advantageous to future clinical applications.

## MATERIALS AND METHODS

### Ethics statement

This project was approved by the Institutional Review Board for Human Research, and was approved by the Ethics Committee of Harbin Medical University, China. All human participants were collected with a written informed consent and were conducted in terms of the Declaration of Helsinki Principles [14].

### Cell culture/sample preparation

MCF-7 cells (Mcfs) was a human breast carcinoma line, which was useful for *in vitro* BC studies because the cell line had retained several ideal characteristics. It was gifted from Professor Liu Feng (Cancer Hospital of Harbin Medical University). Human iPSCs (Hips) were purchased from Innovative Cellular Therapeutics, which were generated by reprogramming human fibroblasts (CCD-1079SK, ATCC) using four transcription factors (TFs) (Oct4, Sox2, Klf4, and c-Myc) delivered by lentiviral vectors. MCFs were cultured in DMEM containing 10% fetal bovine serum (FBS) and 1% penicillin/streptomycin (Invitrogen). Human iPSCs were grown in iPSCs medium (knockout DMEM/F12 plus 20% KOSR, 1% penicillin/streptomycin, 1% NEAA, 1% Glutamax, 100μM β-mercaptoethanol and 10 ng/ml bFGF).

293T cells were transfected with Oct4, Sox2, c-Myc and Klf4 plasmids. After 48h, the viruses supernatant were collected and transduced into MCFs. Then, MCFs were cultured in 10% FBF-DMEM medium for 6 days. At Day7, the induced cells were reseeded on the irradiated mouse embryonic fibroblasts and cultured in iPSCs medium. Cells were subsequently observed for colony formation. About day 20, iPS colonies were isolated and transferred to matrigel-coated plate and maintained in mTeSRTM1 medium (Stem Cell).

### iTRAQ labeling and SCX chromatography

Labeling of samples with iTRAQ and Strong Cation Exchange chromatography (SCX) methods were as previously described [15, 16]. Briefly, the protein samples of Mcfips, MCFs and Hips were reconstituted in dissolution buffer, denatured, reduced, alkylated and then trypsinized. The supernatants were collected, and the total protein concentration was determined using a Bradford protein assay kit. In our study, tryptic digests of Mcfips samples were labeled with 115 and 116 iTRAQ reagents while MCFs samples with 113 and 114 iTRAQ reagents, and Hips samples with 117 and 118 iTRAQ reagents. All samples were balanced, mixed, and were pre-separated using SCX chromatography as described earlier [17, 18]. Then fractions were collected and subjected to LC- mass spectrometer (MS)/MS analysis.

### LC-MS/MS analysis

The fractions were then separated by nano-LC and analyzed by on-line electrospray tandem mass spectrometry. The experiments were performed on a Nano-Aquity UPLC system (Waters Corporation, USA) connected to Triple TOF 5600 MS (Applied Biosystem, USA) [19–22]. The peptide sample was loaded onto the trap column (2.1x 150 mm X Bridge BEH300, Waters Corporation, USA) with a flow of 200 μl/min, and subsequently separated on the analytical column (ZORBAX 300SB-C18 column, 5 μm, 300 Ǻ, 0.1 × 150 mm, microm, USA) with a linear gradient, from 2% D to 80% D in 90 min (solution D: 0.1% formic acid in ACN). The Triple TOF 5600 MS was operated in data-dependent mode to switch automatically between MS and tandem (MS/MS) acquisition. MS spectra were acquired across the mass range of 350–1250 m/z in high resolution mode using 250-millisecond accumulation time per spectrum. Tandem mass spectral scanned from 100–1250 m/z in high sensitivity mode with rolling collision energy. The 20 most intense precursors were selected for fragmentation per cycle with dynamic exclusion time of 9 s.

### Protein identification and relative quantization

The raw data were analyzed using LC MS/MS iTRAQ technology by ProteinPilot™ Software 4.5 (AB Sciex) [15–17]. Protein identification utilized the human SwissProt_2014_08. fasta sequence database. A standard parameter set was used for the search, which included Cys alkylation by methylmethanethiosulfonate (MMTS), biological modifications ID focus, trypsin digestion, Homo sapiens, search effort, and thorough ID. More than two unique peptides were required for protein identification. A threshold of confidence above 95% and a local FDR of less than 1% were used for both protein identification and quantitative analysis [23]. *P*-values < 0.05 were required for relative quantification.

### Bioinformatics analysis

For the subsequent relative quantification analysis, we included an additional FC > 1.3 or < 0.7 (1/1.3)-fold cutoff that was applied to all iTRAQ ratios to reduce false positives for the selection of the DE proteins [24]. The DE proteins were selected by a FDR adjusted *P value* < 0.05. Proteins with iTRAQ ratios below 0.7 were considered to be down-expressed, while those whose ratios were more than 1.3 were deemed to be up-expressed [25].

Functional annotation analysis of the DE proteins was performed using GO (http://www.geneontology.org/) by the online tool DAVID (NIAID, NIH, USA, https://david.ncifcrf.gov/home.jsp) which was classified into three major categories: BP, MF, and CC [26]. The pathway enrichment analysis was performed by KEGG mapping (http://www.kegg.jp/). The annotation with a FDR adjusted *P value* < 0.05 was a considered significant [26].

Functional network construction of protein-protein interactions was performed by Cytoscape 3.4.0 (http://www.cytoscape.org/) [27], using databases HPRD [28], BioGrid [29], and STRING [30]. To obtain the key nodes, various statistical network measures could be calculated, including Degree Centrality, Betweenness, Closeness, and Cluster Coefficient.

## SUPPLEMENTARY MATERIALS FIGURES AND TABLES








