# Supplementary material for: iTRAQ-based quantitative proteomic analysis of Yamanaka factors reprogrammed breast cancer cells
Source: Oncotarget. 2017 Mar 11;8(21):34330–9. doi: 10.18632/oncotarget.16125 (PMC5470971; doi:10.18632/oncotarget.16125)
Supplement: Supplementary file 1 [file oncotarget-08-34330-s001.pdf]

iTRAQ-based quantitative proteomic analysis of Yamanaka-factor reprogrammed breast cancer cells

Supplementary Materials

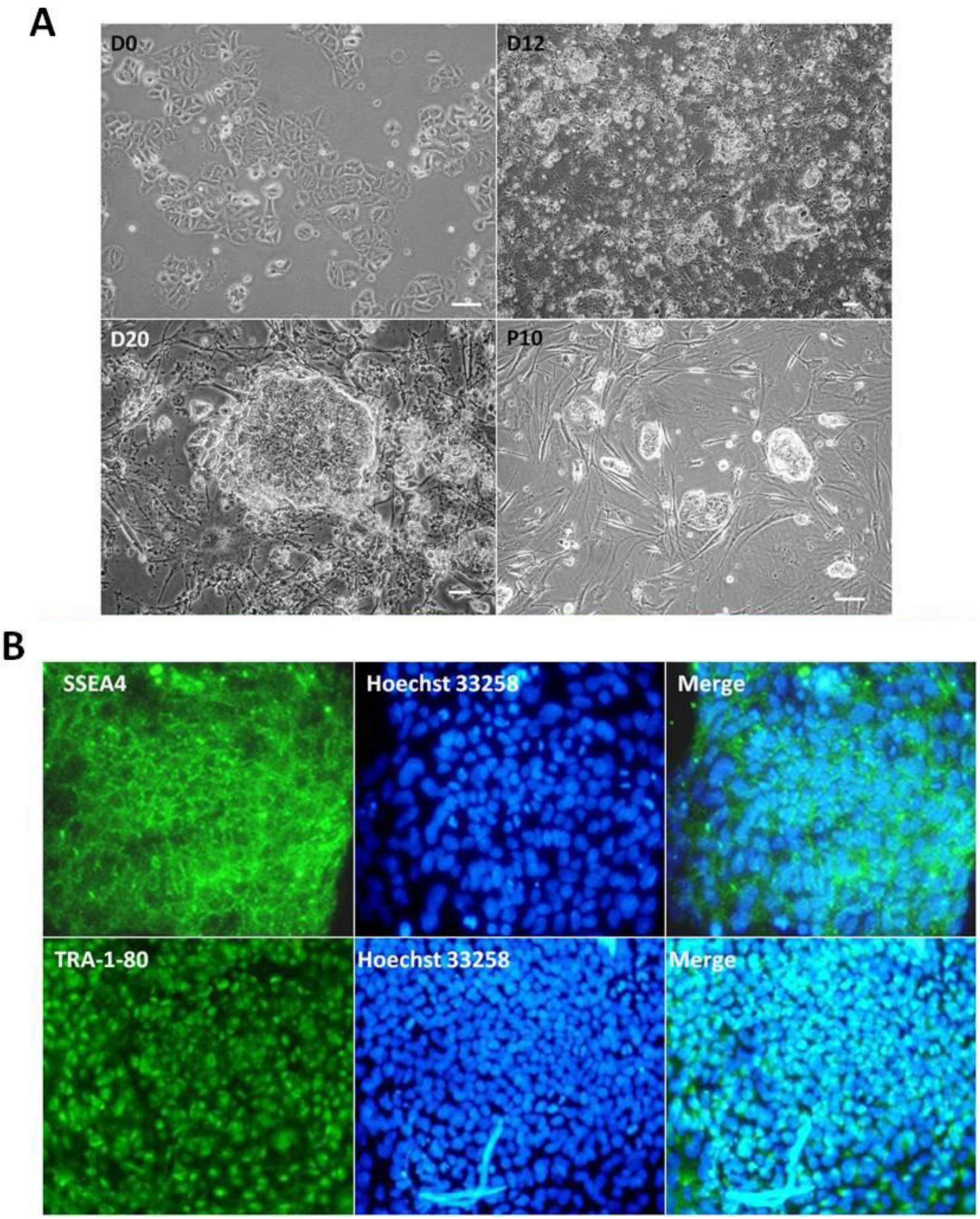

Supplementary Figure 1: Generation of Mcfips by direct delivery of reprogramming. (**A**) Morphology of induced cells at different stage. a, day 0, MCF cells morphology; b, day12, early colonies morphology; c, day20, ES-like colonies morphology; d, P10, were observed for 10 passages. (**B**) The expression of SSEA-4 (green) and TRA-1-80(green) in Mcfips were detected by immunofluorescence. Scale bar, 100 µm.

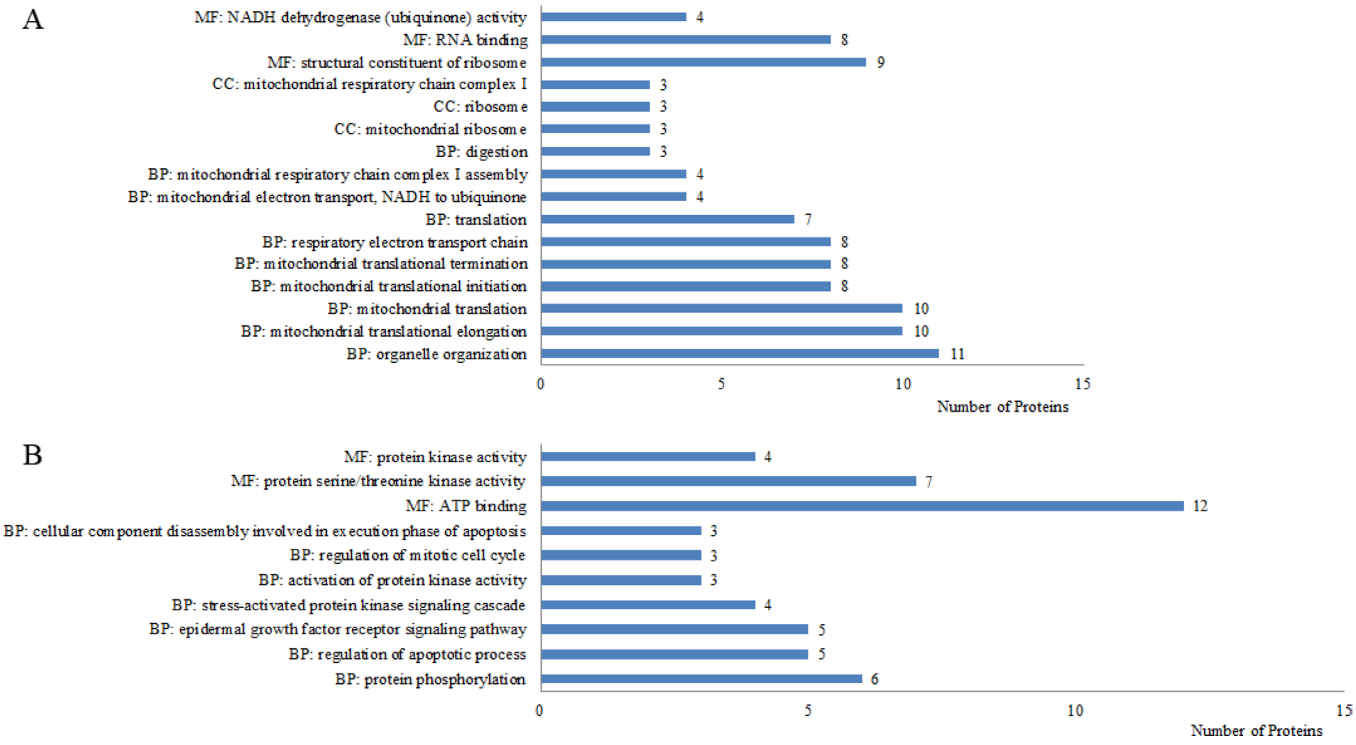

Supplementary Figure 2: Up- (**A**) and down-regulated proteins (**B**) categorized by biological process (BP), molecular function (MF), and cellular component (CC) in Mcfips comparing to MCFs.

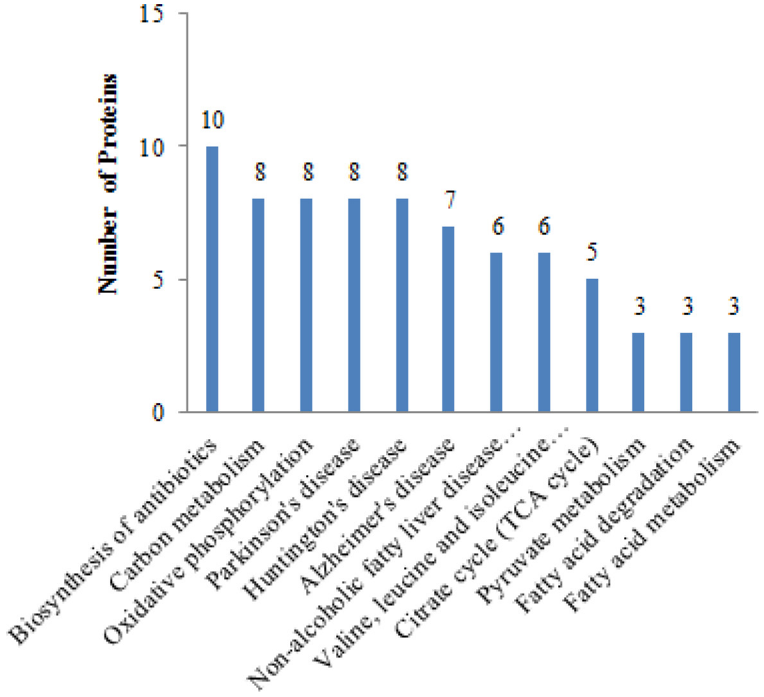

Supplementary Figure 3: Global view of the KEGG pathways affected in Mcfips comparing to MCFs.

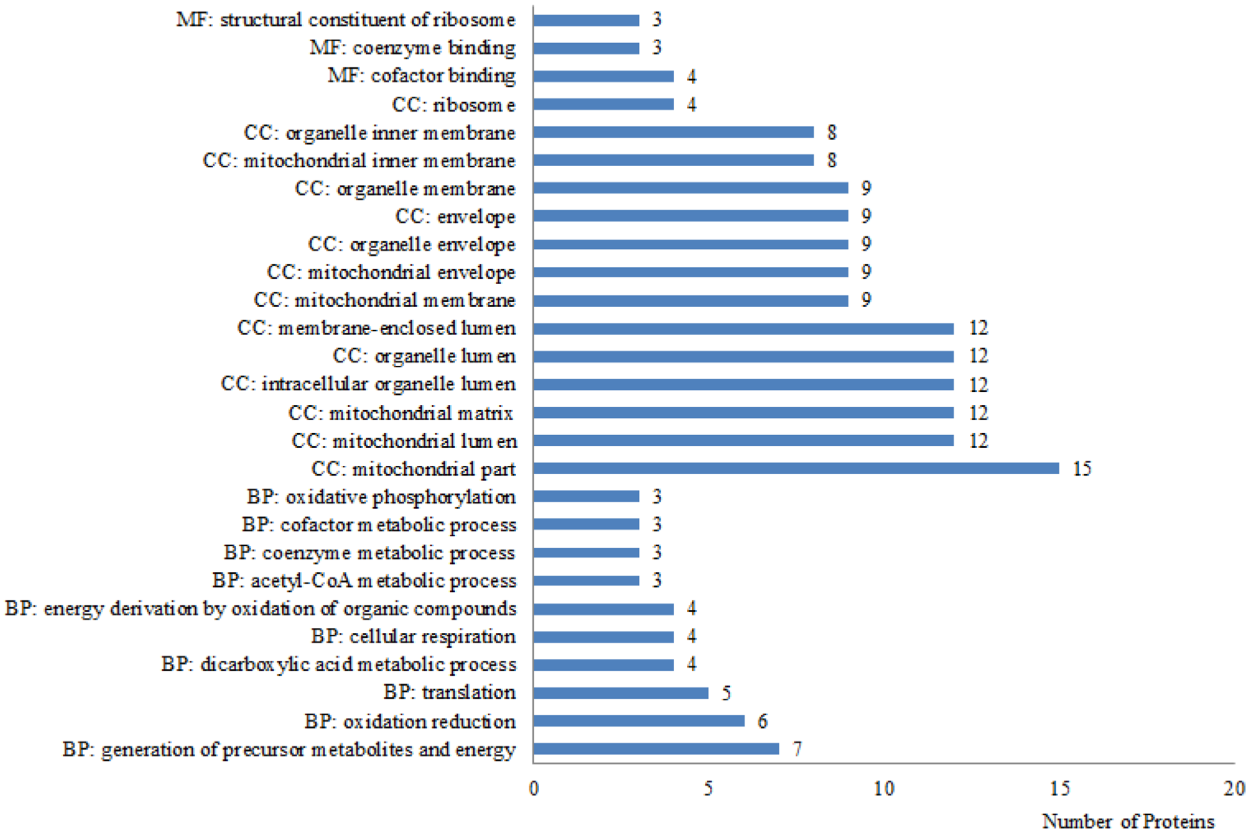

Supplementary Figure 4: Co-up regulated proteins categorized by biological process (BP), molecular function (MF), and cellular component (CC) in Mcfips comparing to Hips and MCFs.

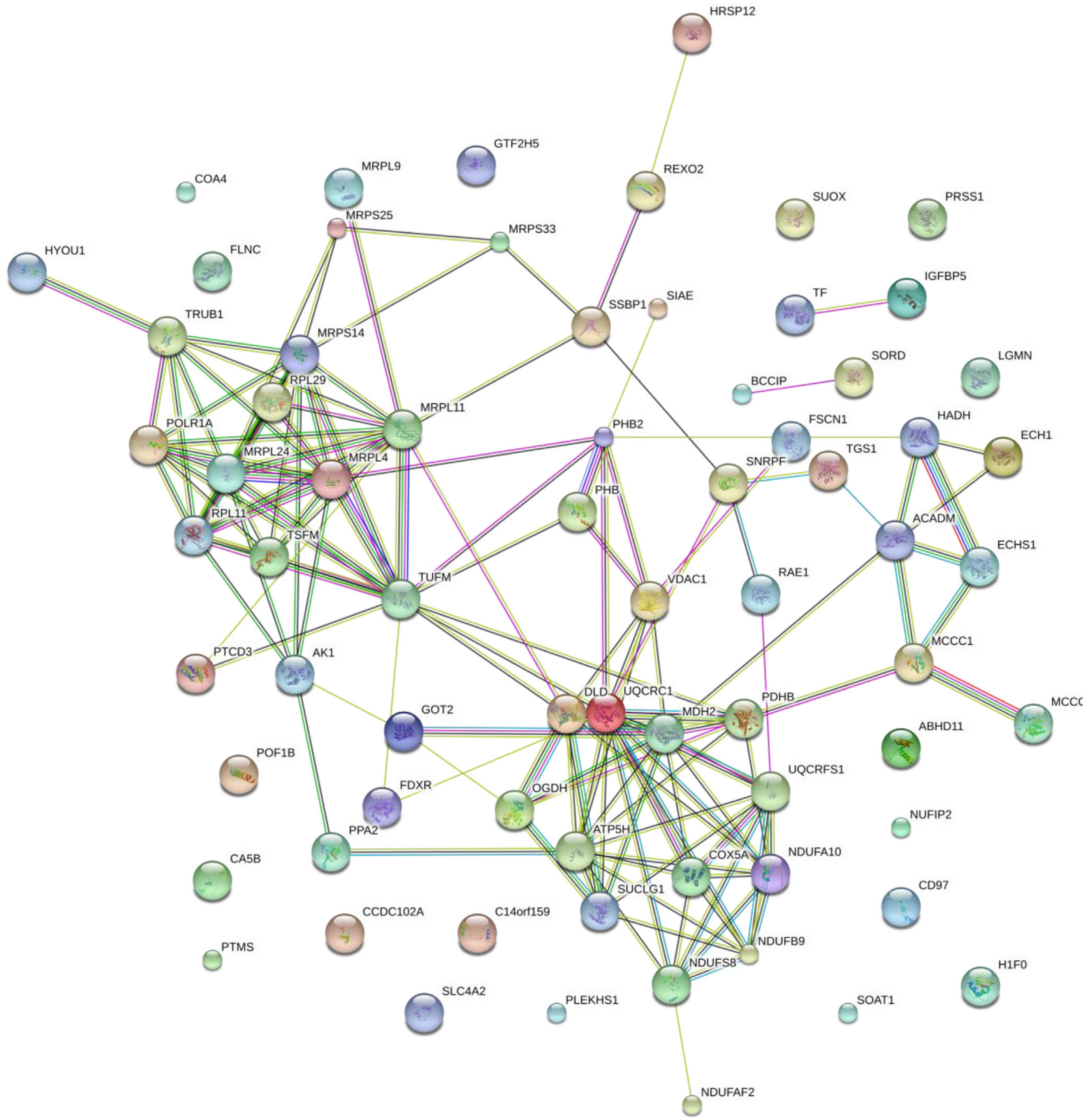

Supplementary Figure 5: Interactome network of up- expressed proteins in Mcfips comparing to MCFs. Coloured lines represent different evidences for each interaction: red line, fusion; green line, neighbourhood; blue line, cooccurrence; purple line, experimental; yellow line, text mining; light blue line, database; black line, coexpression.

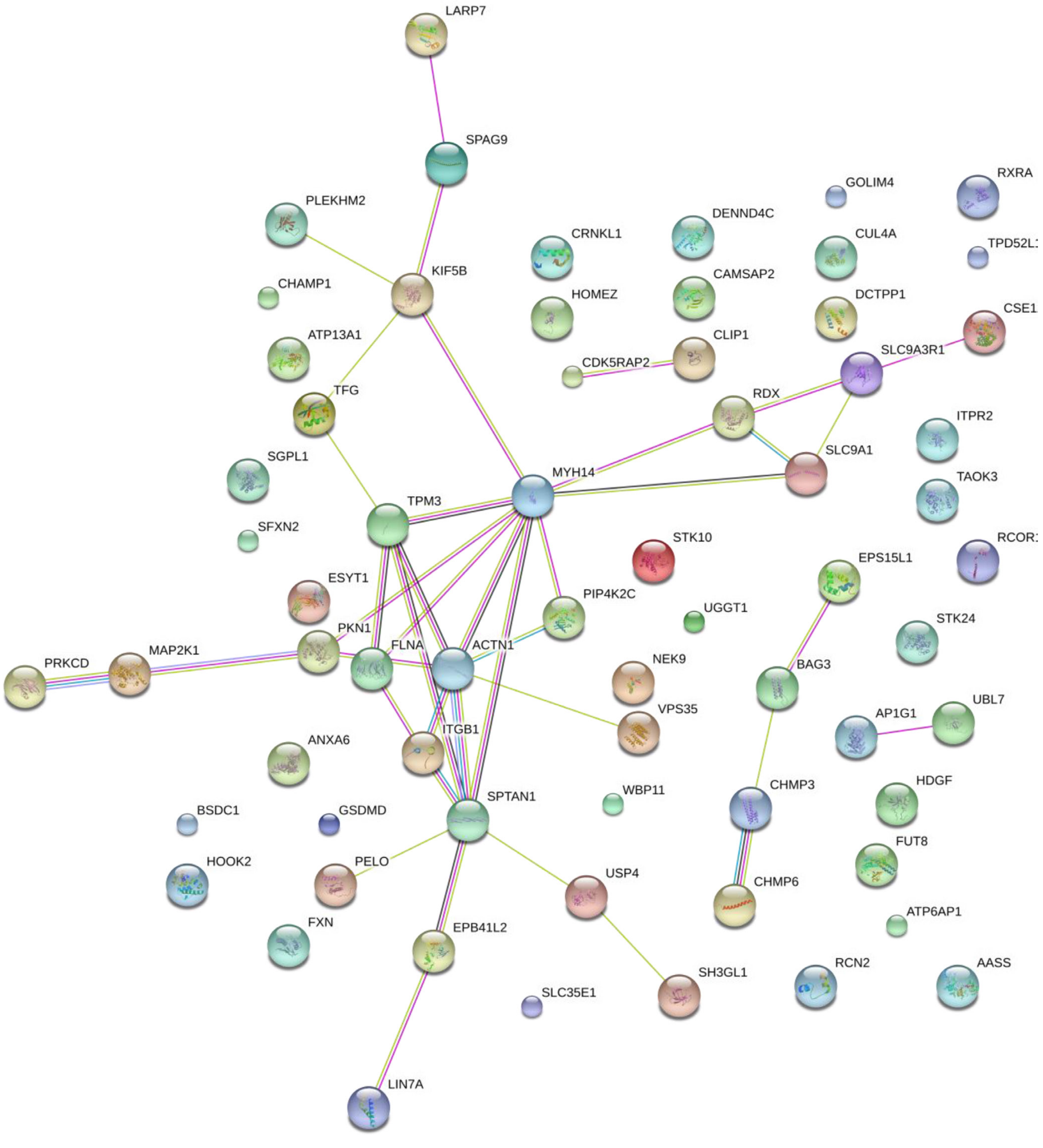

Supplementary Figure 6: Interactome network of down-expressed expressed proteins in Mcfips comparing MCFs. Coloured lines represent different evidences for each interaction: red line, fusion; green line, neighbourhood; blue line, cooccurrence; purple line, experimental; yellow line, text mining; light blue line, database; black line, coexpression.

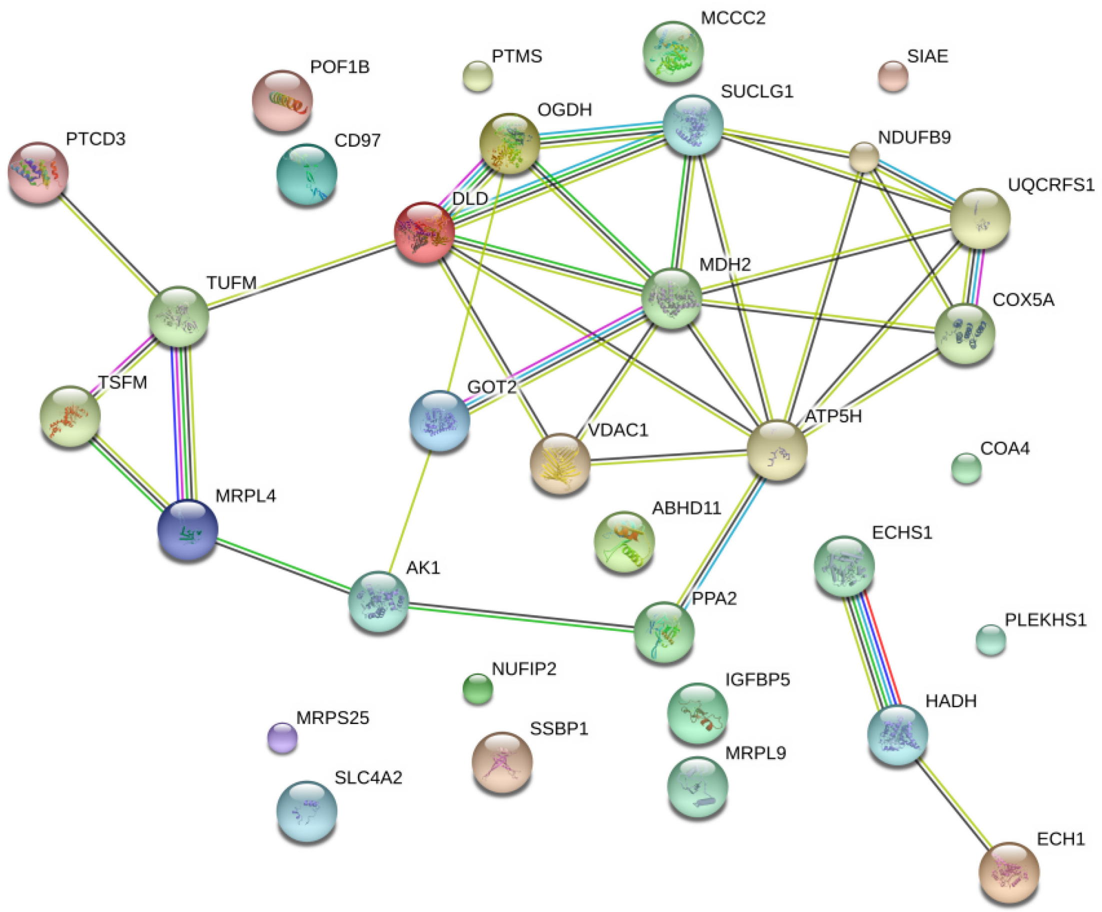

Supplementary Figure 7: Interactome network of 35 co-up expressed proteins in Mcfips comparing to Hips and MCFs. Coloured lines represent different evidences for each interaction: red line, fusion; green line, neighbourhood; blue line, cooccurrence; purple line, experimental; yellow line, text mining; light blue line, database; black line, coexpression.

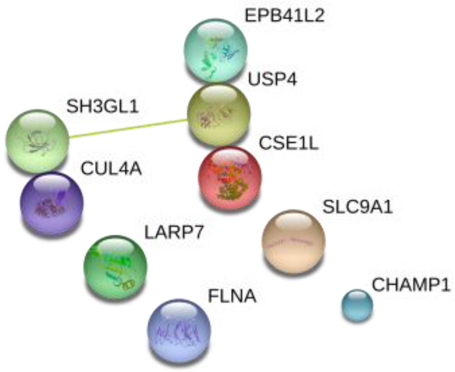

Supplementary Figure 8: Interactome network of 10 co-down expressed proteins in Mcfips comparing to Hips and MCFs. Coloured lines represent different evidences for each interaction: red line, fusion; green line, neighbourhood; blue line, cooccurrence; purple line, experimental; yellow line, text mining; light blue line, database; black line, coexpression.

Supplementary Table 1: The list of proteins found to be expressed at different levels between Mcfips and hips by iTRAQ analysis. See Supplementary_Table_1

Supplementary Table 2: The list of proteins found to be expressed at different levels between Mcfips and MCFs by iTRAQ analysis. See Supplementary_Table_2

Supplementary Table 3: (A) Co-upregulated proteins in Mcfips comparing to hips and MCFs; (B) Co-downregulated proteins in Mcfips comparing to Hips and MCFs; (C) The differentially expressed proteins were up-regulated between Mcfips and Hips, and also were down-regulated between Mcfips and MCFs; (D) The differentially expressed proteins were down-regulated between Mcfips and Hips, and also were up-regulated between Mcfips and MCFs. See Supplementary_Table_3
